# Potential human pathogenic bacteria in five hot springs in Eritrea revealed by next generation sequencing

**DOI:** 10.1371/journal.pone.0194554

**Published:** 2018-03-22

**Authors:** Amanuel Menghs Ghilamicael, Hamadi Iddi Boga, Sylvester Elikana Anami, Tadesse Mehari, Nancy L. M. Budambula

**Affiliations:** 1 Institute for Biotechnology Research, Jomo Kenyatta University of Agriculture and Technology, Nairobi, Kenya; 2 Taita Taveta University, Taita, Kenya; 3 National Commission for Higher Education in Eritrea, Asmara, Eritrea; 4 University of Embu, Embu, Kenya; Oklahoma State University, UNITED STATES

## Abstract

Human pathogens can survive and grow in hot springs. For water quality assessment, *Escherichia coli* or *Enterococci* are the main thermotolerant enteric bacteria commonly used to estimate the load of pathogenic bacteria in water. However, most of the environmental bacteria are unculturable thus culture methods may cause bias in detection of most pathogens. Illumina sequencing can provide a more comprehensive and accurate insight into environmental bacterial pathogens, which can be used to develop better risk assessment methods and promote public health awareness. In this study, high-throughput Illumina sequencing was used to identify bacterial pathogens from five hot springs; Maiwooi, Akwar, Garbanabra, Elegedi and Gelti, in Eritrea. Water samples were collected from the five hot springs. Total community DNA was extracted from samples using the phenol-chloroform method. The 16S rRNA gene variable region (V4—V7) of the extracted DNA was amplified and library construction done according to Illumina sequencing protocol. The sequence reads (length >200 bp) from Illumina sequencing libraries ranged from 22,091 sequences in the wet sediment sample from Garbanabra to 155,789 sequences in the mat sample from Elegedi. Taxonomy was assigned to each OTU using BLASTn against a curated database derived from GreenGenes, RDPII, SILVA SSU Reference 119 and NCBI. The proportion of potential pathogens from the water samples was highest in Maiwooi (17.8%), followed by Gelti (16.7%), Akwar (13.6%) and Garbanabra (10.9%). Although the numbers of DNA sequence reads from Illumina sequencing were very high for the Elegedi (104,328), corresponding proportion of potential pathogens very low (3.6%). Most of the potential pathogenic bacterial sequences identified were from *Proteobacteria* and *Firmicutes*. *Legionella* and *Clostridium* were the most common detected genera with different species. Most of the potential pathogens were detected from the water samples. However, sequences belonging to *Clostridium* were observed more abundantly from the mat samples. This study employed high-throughput sequencing technologies to determine the presence of pathogenic bacteria in the five hot springs in Eritrea.

## Introduction

In Eritrea, there are considerable low temperature-thermal springs that range from 45 to 60 °C and have potential for use as recreation spas, balneotherapy and mineral water bottling. They are located along the Asmara-Massawa highway, close to Gulf of Zula and within the Danakil Depression [1]. A natural thermal pool is a pool with geothermal water that has not been sterilized, irradiated or treated in any way [2]. It is necessary to monitor these geothermal resources in order to provide a safe experience for visitors. Currently, there is no legislation or guideline in place regarding the use of hot springs in Eritrea. Therefore, the public use of hot springs in Eritrea cannot be regarded as safe. Human pathogens can survive and grow in hot springs. A vast diversity of microorganisms, originating from different sources, such as humans, animals or the environment, have been found in pools and other waters used for recreation [3–6].

*E*. *coli* or *Enterococci* are the main thermotolerant enteric bacteria invariably used to enumerate the load of pathogenic bacteria in water [7,8]. Doubts have been raised on the reliability of *E*. *coli* or other coliform bacteria in describing the pathogenic potential of a water body due to low levels of correlation with the presence of pathogens and low sensitivity of detection methods [9]. Most of the environmental bacteria are unculturable [10]. Moreover, culture methods may cause bias in detection of most pathogens [11]. Molecular technologies, such as polymerase chain reaction (PCR), quantitative real time PCR and microarrays have been widely used to detect pathogens in sewage and natural waters [12,13]. However, these methods only detect specific pathogens and may not provide a comprehensive insight of potential pathogens in the environment. High-throughput sequencing technologies can provide exhaustive sequencing depth to cover the broad microbial community in the environment [14]. Next generation sequencing (NGS) platform allows the identification and characterization of microbial community members in an ecosystem at a depth of up to millions of sequences per sample promoting the identification of microorganisms present in low numbers [15,16]. The 16S rRNA gene Illumina NGS with universal bacterial primers has been reported to be more effective at screening pathogens than 16S rRNA denaturing gradient gel electrophoresis (DGGE) combined with the subsequent sequencing of DGGE bands [15].

This study investigated the presence of pathogens in the five hot springs in Eritrea using Illumina high-throughput sequencing.

## Materials and methods

### Research authorization

Research authorization was obtained from National Board for Higher Education on 2^nd^ November, 2013 in Eritrea (which has since been renamed the National Commission for Higher Education). Permission to undertake research in the Eritrean hot springs was granted by the Ministry of Land, Water and Environment, Eritrea on 5^th^ of December, 2013.

### Study site

The Samples analyzed in this study were collected from five hot springs (Table 1).

**Table 1:** Location and physical characteristics of the five hot springs in Eritrea.

Location	Latitude °N	Longitude °E	Hot spring	Elevation (m)	T (°C)	pH	Salinity (%)
Alid area	14°52'55''	39°54'34''	Elegedi	515.0	100	7.19	0.9
Near Gahtelai	15°33'34''	39°05'47''	Akwar	344.51	49.0	6.97	0.7
Near Gahtelai	15°32'53''	39°06'38''	Maiwooi	330.10	51.9	7.54	0.4
Near Irafayle	15°03'29''	39°46'27''	Garbanabra	0.00	51.0	7.05	3.2
Near Irafayle	15°03'39''	39°46'46''	Gelti	0.00	52.7	7.01	3.1

Akwar and Maiwooi are low energy hot springs which discharge near-neutral bicarbonate waters [1]. Garbanabra and Gelti are located near Irafayle on the shore of Gulf of Zula. The salinity (3.2 and 3.1%, respectively) is much higher than Maiwooi and Akwar (0.4 and 0.7%, respectively) due to mixing with the sea water. Elegedi, the boiling hot spring located in Alid volcanic center about 30 km south of the Gulf of Zula, is associated with a high temperature geothermal system underlying the Alid volcanic centre in the Northern Danakil depression of Eritrea [1]. The bubbling water discharged from this hot spring is typical of the fumarolic steam condensate with high temperatures.

### Sample collection and preparation

From each hot spring, triplicate samples of water, wet sediment and microbial mat were collected. The water, wet sediment and microbial mat samples collected from each hot spring were stored at -80 °C until DNA extraction was done. Approximately 500 g of wet sediment samples were collected from the upper sediment and placed in 500 ml autoclaved bottles by scooping using sterile spoon. Mat samples were aseptically scraped using a sterile spatula into sterile zip lock plastic bags. All samples were preserved on dry ice immediately after sampling and transported to the Quality Control Laboratory (QCL) in Massawa, Eritrea

Community DNA was extracted from water, sediment and microbial mat samples as described by Kambura et al [17]. From each wet sediment and microbial mat samples 0.5 g were placed in 1.5 ml Eppendorf tubes. Approximately 500 ml of the water samples was filtered through 0.22 μm Whatman filter papers and centrifuged. All water samples formed a visible pellet upon centrifugation. The pellet, from each of the water samples, was re-suspended in 5 ml of phosphate-buffered saline solution. After three successive washings in phosphate-buffered saline solution, the pellet was placed in 1.5 ml Eppendorf tubes. DNA was extracted from each sample using the phenol chloroform method [18]. Extracted DNA pellets were air dried and stored at -20 °C.

### Amplicon library preparation

PCR amplification of the 16S rRNA gene V4-V7 variable region was carried out from extracted DNA using barcoded primers 515F (GTGCCAGCMGCCGCGGTAA) and 806R (GGACTACHVGGGTWTCTAAT) as previously described [19]. PCR amplification was carried out in 30 cycles using the HotStarTaq Plus Master Mix Kit (Qiagen, USA) under the following conditions: 94°C for 3 minutes of initial heating, followed by 30 cycles of 94°C for 30 seconds, 53°C for 40 seconds and 72°C for 1 minute, after which a final elongation step at 72°C for 5 minutes was performed. The quality of PCR products was assessed on a 2% agarose gel to determine the success of amplification and the relative intensity of bands. Multiple samples, tagged with different barcodes, were pooled together in equimolar ratios based on their DNA concentrations from the gel images. Pooled samples were purified using calibrated Ampure XP beads (Beckman Coulter) for use in library preparation. The pooled and purified PCR product was used to prepare the DNA library by following Illumina TruSeq DNA library preparation protocol [20]. Sequencing was performed at Molecular Research DNA (www.mrdnalab.com, Shallowater, TX, USA) on a MiSeq 2x300bp Version 3 following the manufacturer’s guidelines.

### Sequence analysis, taxonomic classification and data submission

Sequences obtained from the Illumina sequencing platform were depleted of barcodes and primers using a proprietary pipeline (Molecular Research DNA, Shallowater, Texas) developed at the service provider’s laboratory. Low quality sequences were identified by denoising and filtered out of the dataset [21]. Short sequences < 200 base pairs after phred20- based quality trimming, sequences with ambiguous base calls, and those with homopolymer runs exceeding 6 bp were removed. Sequences were analyzed by a script optimized for high-throughput data to identify potential chimeras in the sequence files. All definite chimeras were depleted as previously described [22]. Operational taxonomic units (OTUs) were defined by clustering at 3% divergence (97% similarity). All data filtering was done by the service provider using their pipeline. Taxonomy was assigned to each OTU using BLASTn against a curated database derived from GreenGenes, RDPII, SILVA SSU Reference 119 and NCBI [23]. Resulting raw sequences were submitted to NCBI Sequence Read Archive with study accession number SRP064297.

### Statistical analysis

Rarefaction curve was calculated from the resulting OTUs using Vegan package version 1.16–32 in R software version 3.1.3 [24]. Hierarchical clustering of the sequences assigned to potential pathogens at species level, based on Bray-Curtis dissimilarity, was carried out using the R programming language [24] and the Vegan package [25].

## Results

### Taxonomic assignment of the sequences

**Fig 1:**
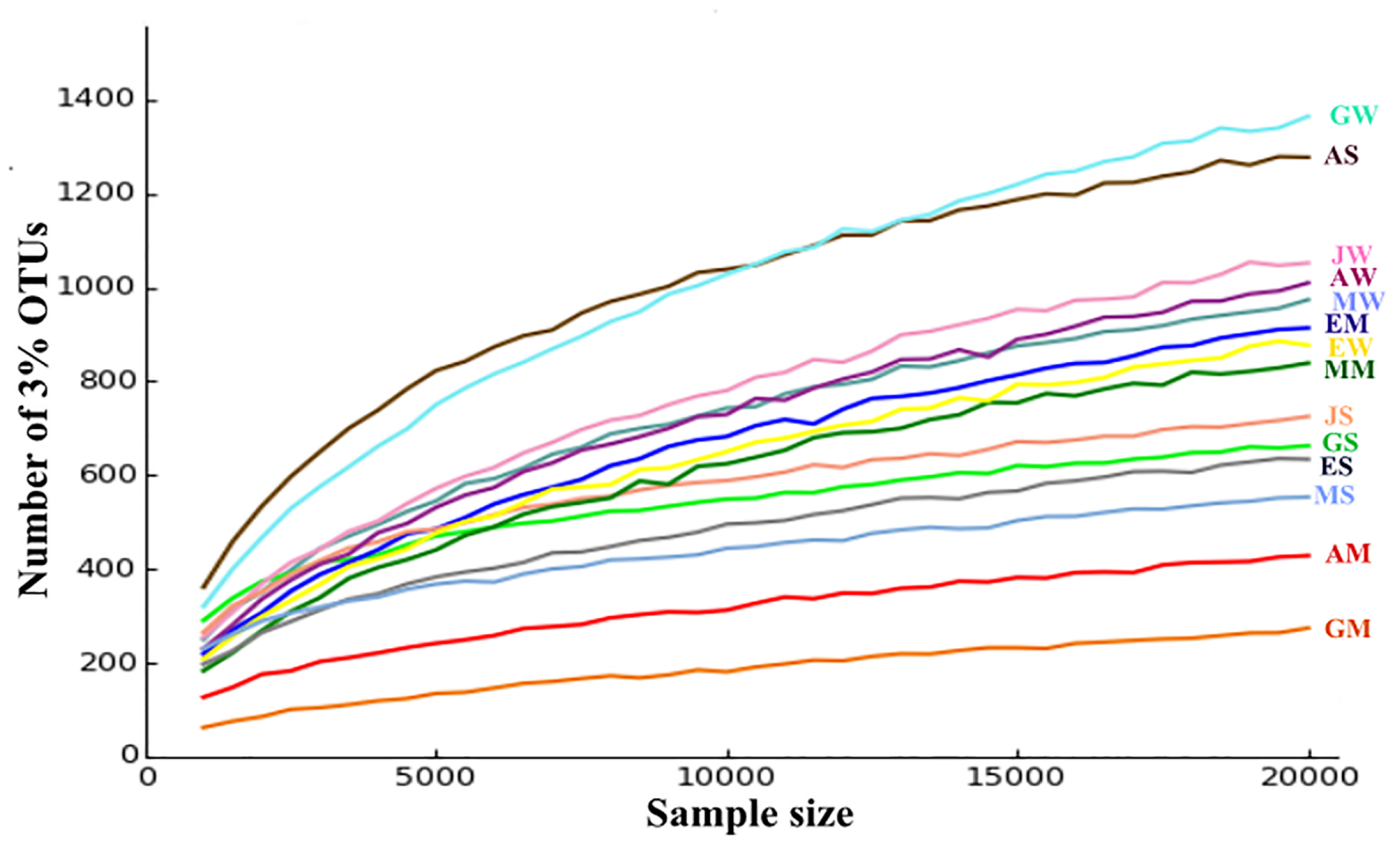
Rarefaction curves of OTUs at cutoff of 3% from amplicon samples collected from the five hot springs in Eritrea. The first letters of the sample names refer to the five hot springs (A = Akwar, E = Elegedi, G = Garbanabra, J = Gelti and M = Maiwooi), while the second letters are for sample types (A = Microbial mat, S = wet sediment, and W = water).

The sequence reads (length >200 bp) from Illumina sequencing libraries ranged from 25,586 sequences in Maiwooi to 113,743 sequences in Akwar. The reads contained between 1,662 OTUs and 2,354 OTUs, respectively. Rarefaction curve was plotted in order to evaluate if all the diversity within the hot springs have been exhaustively recovered (Fig 1). From the rarefaction curves generated, analyses suggest that sequencing could be expanded in order to ensure the inclusion of the entire diversity of the prokaryotes in these hot springs

A compilation of reference human pathogenic bacteria list, including the species and genus names, disease caused, and the risk group (RG) was done using the sequences of known pathogens and National Institute of Health, USA (NIH) Appendix B in order to identify the potentially pathogenic bacterial sequences from the large amount of sequences [26]. The sequences obtained in this study were first assigned at the genus level using SILVA SSU Reference 119. Sequences showing high similarity with human pathogenic bacteria were extracted and the individual sequences were searched using online BLAST search (http://blast.ncbi.nlm.nih.gov/blast) in order to confirm and double-check the assignment results of the SILVA SSU Reference 119.

### Potential pathogenic bacteria

Members of at least 30 bacterial phyla were detected using Illumina sequencing. Most of the potentially pathogenic bacterial 16S rRNA encoding DNA sequences were identified from the *Proteobacteria* and *Firmicutes*. The number of sequences identified as potentially pathogenic bacteria, at genus level, using Illumina sequencing in all samples is shown in S1 Table. The genera *Pseudomonas*, *Bacillus*, *Aeromonas*, *Clostridium*, *Legionella*, *Escherichia*, *Burkholderia* and *Acinetobacter* occurred in all the hot springs investigated. However, some of these may be at the risk group 1 level or opportunistic pathogens. Relatively higher abundance of the potential pathogenic bacteria were observed from the mat samples. The highest percentage was recorded from the mat sample of Garbanabra (41%). From water samples, the highest relative abundance of the potential pathogenic bacteria was observed in Maiwooi (17.8%) followed by Gelti (16.7%), Akwar (13.6%) and Garbanabra (10.9%). The boiling (100 °C) hot spring Elegedi had the lowest relative abundance of the potential pathogenic bacteria (4.2%). The most dominant genus was *Pseudomonas*, although this did not include any of the highly recognized pathogenic species *Legionella*, the second most abundant genus, was recovered abundantly from the water samples of Maiwooi, Akwar and Gelti.

The potentially pathogenic bacteria were identified by BLAST to the species level and are presented in S2 Table. Among the 15 pathogen genera considered (Table 2), there were sequences of 27 species that are classified as potential pathogens with risk group 2 ratings according to NIH Appendix B: Classification of human etiologic agents on the basis of hazard, 2016.

**Table 2:** Potential human pathogenic bacterial sequences identified from five hot springs in Eritrea.

Genus	Species	Source*	Risk group	Diseases	Percent similarities	Accession number
***Aeromonas***	*hydrophila*	All	2	Gastroentreritis	99	NC_0085701.1
***Acinetobacter***	*johnsonii*	All	2	Nosocomial	99	NZ_CP010350.1
***Legionella***	*Pneumophila*	All	2	Legionnaires’ Disease, Pontiac fever	99	NR_074231.1
	*Oakridgensis*	A,J,M	2	Pneumonia	99	NR_1211730.1
	*Gresilensis*	All	2	Pneumonia	99	NR_028744.1
	*Drozanskii*	A,E,G,J	2	Pneumonia	97	NR_036803.1
	*Lytica*	A	2	Pneumonia	99	NR_026334.1
	*jordanis*	J,M	2	Pneumonia	98	NR_036992.1
	*iondiniensis*	A,E,G,J	2	Nonpathogenic	96	NR_044963.1
	*dresdenensis*	A,G,J,M	2		96	NR_115062.1
	*rubrilucens*	All	2		96	NZ_LNYT010000022.1
	*maceachernii*	A,E,J	2		97	NR_041790.1
***Brucella***	*melitensis*	All	3	Brucellosis	97	NR_104538.1
	*papionis*	A,J	3	Brucellosis	97	NR_133990.1
***Burkholderia***	*Cepacia*	All	2	Cystic fibriosis	99	NZ_CP012983.1
***Clostridium***	*botulinum*	A,E	2	Botulism	99	NR_029260.1
	*Tetani*	A,E,J	2	Tetanus	98	NR_029260.1
	*tunisiense*	A,E,G,J	2	Nonpathogenic	96	NZ_AMQHO1000037.1
	*tetanomorphum*	A	2	Nonpathogenic	99	NR_043671.1
	*homopropionicum*	A,E,G,J	2	Nonpathogenic	98	NR_026148.1
	*Pascui*	A	2	Nonpathogenic	99	NR_026322.1
	*saccharobutylicum*	A,G	2	Nonpathogenic	99	NR_122051.1
***Corynebacterium***	*diphtheriae*	All	2	Respiratory disease	99	NR_119135.1
	*tuberculostearicum*	A,E,G,J	2	Granulomatous mastitis	99	NR_028975.1
***Escherichia***	*Coli*	All	2	Diarrheal disease	99	NR_114079.1
***Haemophilus***	*parainfluenzae*	G	2	Infective endocarditis	99	NR_116168.1
***Moraxella***	*Osloensis*	A,G,M	2	Conjunctivitis	99	NR_113392.1
***Streptococcus***	*Gordonii*	J,M	2	Nonpathogenic	99	NR_116205.1
	*equinus*	A,G	2		99	NR_113594.1

*A = Akwar, E = Elegedi, G = Garbanabra, J = Gelti and M = Maiwooi

Based on Illumina sequencing, potential pathogens belonging to risk group 1, risk group 2 and risk group 3 were detected. The most common potentially pathogenic species from the genus *Legionella* was *Legionella pneumonia* which was isolated from all the five hot springs. Two sequences that were 97% similar to *Brucella melitensis* and *Brucella papionis*, which are both risk group 3 pathogens, were also detected. Sequences belonging to *B*. *melitensis* were recovered from all the five hot springs while those of *B*. *papionis* were recovered from Akwar and Gelti.

### Pathogenic bacteria composition

**Fig 2:**
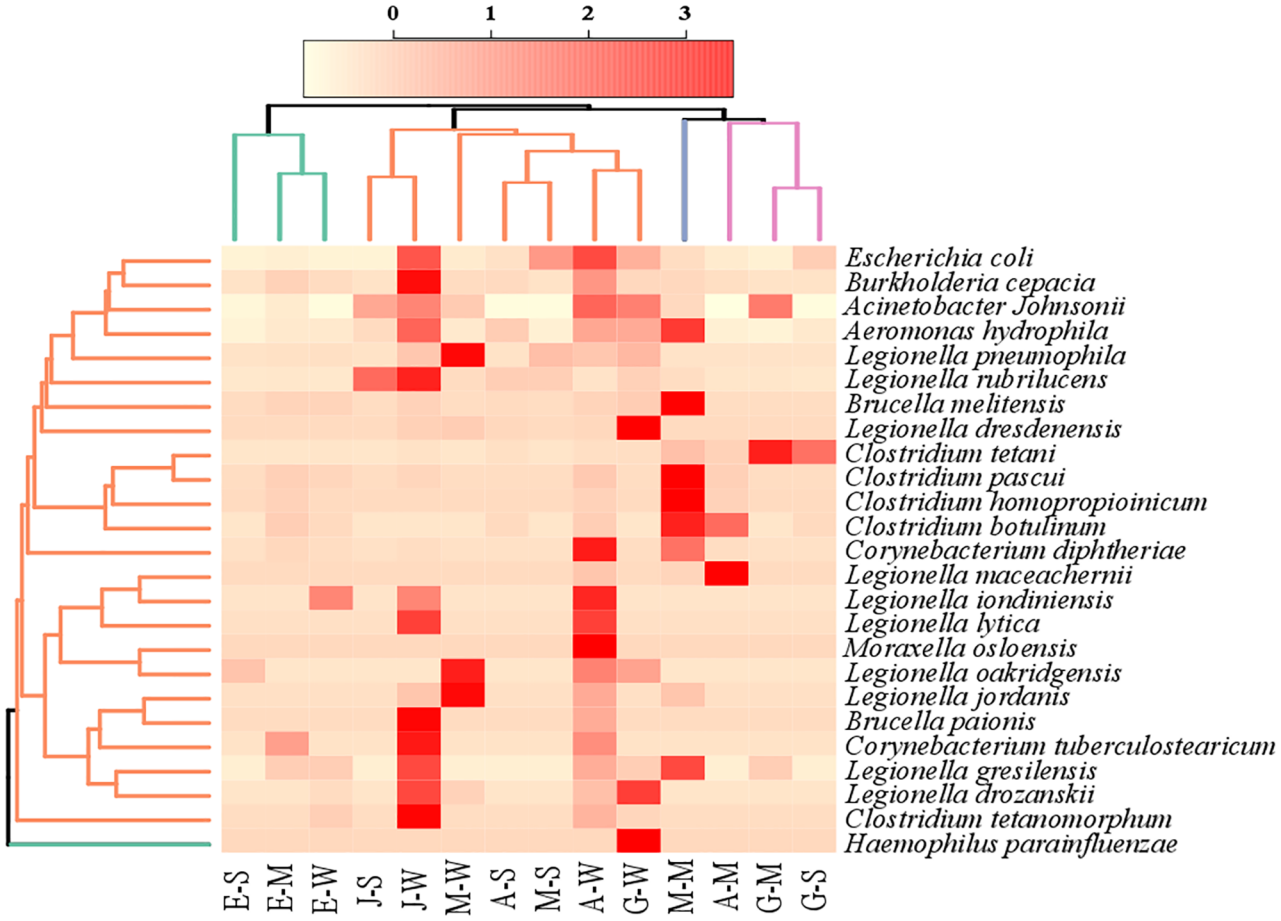
Hierarchical clustering, based on Bray-Curtis dissimilarities, illustrating the composition of potential pathogens at species level in the five hot springs in Eritrea. The first letters of the sample names refer to the five hot springs (A = Akwar, E = Elegedi, G = Garbanabra, J = Gelti and M = Maiwooi), while the second letters are for sample types (A = Microbial mat, S = wet sediment, and W = water).

Hierarchical clustering, based on Bray-Curtis dissimilarity, of the potential pathogens at species level is shown in Fig 2.

Elegedi, the boiling hot spring, was shown to form a separate cluster. Analysis of similarities (ANOSIM) between the hot springs revealed significance (r = 0.53, p = 0.003). This suggested that Elegedi had significantly low numbers of sequences belonging to potential pathogens at species level compared to other hot springs. *L*. *pneumophila*, *Legionella oakridgensis* and *Moraxella osloensis* were abundant in the water samples of Maiwooi. In the water samples obtained from Gelti, *Legionella rubrilucens* (845 sequence reads), *Legionella drozanskii* (32 sequence reads), *Legionella jordanis* (9 sequence reads) and *Legionella taurensis* (4 sequence reads) were more abundant. *Burkholderia cepacia* (101 sequence reads), *Aeromonas hydrophila* (46 sequence reads) and *Corynebacterium tuberculostearicum* (5 sequence reads) were also detected at relatively high abundance from Gelti. *Legionella dresdensis* (60 sequence reads) was recovered at relatively higher numbers from Garbanabra than from the other hot springs. *Haemophilus parinfluenza* (2 sequence reads) was detected only from water samples of Garbanabra. Most of the sequences belonging to genus *Clostridium* were detected mainly from the mat samples of Akwar, Maiwooi and Garbanabra. *Legionella lytica* was detected from the water samples of Akwar and Gelti. *L*. *dresdensis* (60 sequence reads) was most abundant in water sample obtained from Garbanabra. Sequences affiliated to *E*. *coli* were detected at relatively high abundance from Akwar (88 sequence reads) and Gelti (82 sequence reads). *B*. *cepacia* was recovered at a relatively high abundance from the two saline hot springs, Gelti (101 sequence reads) and Garbanabra (36 sequence reads).

Based on ANOSIM, significant difference (r = 0.24, p = 0.047) was observed in the distribution of the sequences belonging to the potential pathogens between the sample types. Most of the sequences belonging to pathogenic bacteria were obtained from the water samples. Sequence affiliated to *Clostridium* were recovered mainly from the mat samples.

## Discussion

The presence of potential pathogens in Garbanabra, Maiwooi, Akwar and Gelti could be a public health concern. Maiwooi and Akwar are frequently visited by many people as they are conveniently located between the capital city, Asmara and the town of Massawa. Therefore, the public health issue is more pronounced in these two hot springs. Elegedi, with a temperature of 100 °C, had few sequences affiliated with human pathogens. This hot spring is too hot and too far to be used for purposes of recreation and health. The data on Elegedi were included in the present investigation for comparison. Most of the pathogens detected were obtained from the water samples. However, sequence reads belonging to the genus *Clostridium* were more abundant in the mat samples. The abundance of *Clostridium* from mat samples has been previously reported from Lake Fryxell, Antarctica [27]. The authors reported that more than 10% of the cloned 16S rRNA gene sequences and five of the isolates obtained belonged to the genus *Clostridium*.

Human pathogenic bacteria have been reported in Sungai Klah, the second hottest hot spring in Malaysia with temperatures varying from 50 to 110 °C [28]. The study reported the presence of sequence reads affiliated to pathogenic bacteria such as; *Clostridium difficile*, *Clostridium hiranonis*, *Brucella suis*, *L*. *pneumophila*, *Leptospira licerasiae*, *Leptospira wolffii*, *Pseudomonas fluorescens*, *Rickettsia montanensis*, *Rickettsiales* and others.

In the present study, *Legionella* and *Clostridium* were the most commonly detected genera represented by different species. Water from the air-conditioning cooling systems, condensed water system, cold and hot water supply systems and hot springs have been implicated as main sources of infection causing outbreaks and legionellosis epidemics [29]. Outbreaks and cases of *Legionella* infections that are associated with hot springs have been previously reported [30]. The occurrence of legionellosis has been reported in several countries. There are 8000–18,000 cases of legionellosis annually in the United States [31]. In Japan, the frequency is much lower, around 20 cases a year during the last few years [32]. *L*. *pneumophilia* at a sequence similarity of 99% was the most common potentially pathogenic bacteria that was detected from all the five hot springs in Eritrea. Other species of *Legionella* detected in the present study include; *L*. *oakridgensis*, *L*. *grasilensis*, *L*. *drozanskii*, *L*. *lytica*, *L*. *jordanis*, *L*. *iondiniensis*, *L*. *dresdenensis*, *L*. *rubrilucens* and *L*. *maceachernii*. The distribution of *Legionella* from seven hot spring recreational areas throughout Taiwan also showed the predominance of *L*. *pneumophila* (25%) in the samples, while *L*. *bozemanii*, *L*. *dumoffi*, *L*. *feelei*, *L*. *lyticum* and *L*. *oakridgenesis* were all detected once [33]. *Legionella* is able to reproduce at temperatures between 25 and 50 °C and survive in temperatures of up to 55–60 °C [34]. Therefore, it can be found in naturally and artificially hot water systems. The pH of all spring water samples in the present study were near 7, which is an optimal pH for *Legionella* in an aquatic environment [35]. Many *Clostridium* species have been implicated as human pathogens [36,37]. Neurotoxigenic clostridia such as *C*. *botulinum* and *C*. *tetani* were detected mainly from Akwar.

Sequences showing 99% similarity to mesophilic *A*. *hydrophila* were detected from all the hot springs (with a maximum abundance of 46 at Gelti). *Aeromonas* species have been found in a variety of aquatic environments including; lakes, rivers, streams, springs, rainwater, swimming pools and seawater [38]. In humans, infections caused by *Aeromonas* species generally result in either acute or chronic gastrointestinal illness, septic arthritis, or water/soil associated traumatic wound infections [39,40]. *Aeromonas* wound infections are commonly caused by *A*. *hydrophila* and have been reported after accidental puncture of the skin followed by exposure to contaminated water or soil [41,42]. Sequences with a 99% similarity to *B*. *cepacia*, were more prevalent in the two saline hot springs, Garbanabra and Gelti. *B*. *cepacia* has been implicated for causing fatal necrotizing pneumonia and bacteremia, especially in patients with cystic fibrosis or chronic granulomatous disease [43,44].

In Garbanabra and Gelti, sequences showing 99% similarity to *E*. *coli* were predominantly detected. *E*. *coli* and *Enterococcus* are invariably used as indicators of fecal contamination and other potential intestinal bacteria. *E*. *coli* and *Enterococcus* were previously detected using culture techniques in pools at Hveravellir and Landmannalaugar which are presumed to be the most visited natural thermal pools in Iceland [2]. Surprisingly, Maiwooi, most frequently visited hot springs in Eritrea, had relatively lower numbers of *E*. *coli* compared to Akwar, Garbanabra and Gelti.

Two sequences that were 97% similar to *Brucella* were identified in this study. These are risk group 3 agents that are associated with serious or lethal human disease. Preventive or therapeutic interventions may be available for risk group 3 in comparison to risk group 4 agents for which preventative or therapeutic interventions are not available. They are the causative agents of brucellosis (undulant fever or Malta fever). In this study *Brucella melitensis* was detected from all the hot springs except Elegedi. Recently, *B*. *suis* was detected from the second hottest Sungai Klah hot spring (50–110 °C) in Malaysia [28]. *Brucella spp*. infects humans as an incidental host and infection usually results from direct contact with tissues or blood from infected animals or polluted recreational water as well as by consumption of animal products, including unpasteurized milk and cheese [45]. *Brucella spp*. are serious animal pathogens that cause brucellosis, a zoonosis that results in substantial economic losses, human morbidity and perpetuates poverty worldwide [46].

## Conclusion

The main objective of this study was to identify potential pathogens in the five hot springs from Eritrea. These potential pathogens detected using NGS could be enriched and studied or quantified using real-time PCR after designing primers at the genus level to focus on the quantification of pathogens with potential risk to human health. This could lead to better understanding of pathogen load in the environment.

This study provided a comprehensive insight into bacterial pathogens and demonstrated the applicability of the high-throughput sequencing technologies in detection of human pathogens from environmental samples. To our knowledge, this is the first study which employed high-throughput sequencing technologies to investigate pathogenic bacteria in the five hot springs in Eritrea. Technical complementation of the Illumina sequencing can provide a more comprehensive and accurate insight into bacterial pathogens in the environment. High-throughput sequencing technologies will serve as a powerful and promising approach to monitor and track human bacterial pathogens. However, compared with culture-based methods, the molecular technologies have some limitations on specificity, since pathogenicity may vary among the different strains within one species [47]. For example, adhesiveness, invasiveness, cytotoxicity and diarrhea infectivity of *Arcobacter butzleri* strains varied among different animal hosts [48]. The difficulties incurred in quantifying pathogens in terms of cell number in surface water using such molecular technique should also be underlined [26].

## Supporting information

S1 Table Number of sequence reads assigned to potential pathogenic genus from the water samples of five hot springs in Eritrea as determined by Illumina sequencing using SILVA SSU Reference 119. The first letters of the sample names refer to the five hot springs (A = Akwar, E = Elegedi, G = Garbanabra, J = Gelti and M = Maiwooi), while the second letters are for sample types (A = Microbial mat, S = wet sediment, and W = water).
(DOCX)

S2 Table Abundance of potential pathogens at species level in various samples collected from the five hot springs in Eritrea. The first letters of the sample names refer to the five hot springs (A = Akwar, E = Elegedi, G = Garbanabra, J = Gelti and M = Maiwooi), while the second letters are for sample types (A = microbial mat, S = wet sediment, and W = water).
(XLSX)
