# Supplementary material for: Potential human pathogenic bacteria in five hot springs in Eritrea revealed by next generation sequencing
Source: PLoS One. 2018 Mar 22;13(3):e0194554. doi: 10.1371/journal.pone.0194554 (PMC5864021; doi:10.1371/journal.pone.0194554)
Supplement: S1 Table — The first letters of the sample names refer to the five hot springs (A = Akwar, E = Elegedi, G = Garbanabra, J = Gelti and M = Maiwooi), while the second letters are for sample types (A = Microbial mat, S = wet sediment, and W = water). (DOCX) [file pone.0194554.s001.docx]

| **Genus** | **Hot Spring** | | | | | | | | | | | | | | **Total** |
| --- | --- | --- | --- | --- | --- | --- | --- | --- | --- | --- | --- | --- | --- | --- | --- |
|  | **Akwar** | | | **Elegedi** | | | **Garbanabra** | | | **Gelti** | | **Maiwooi** | | |  |
|  | **M** | **WS** | **W** | **M** | **WS** | **W** | **M** | **WS** | **W** | **WS** | **W** | **M** | **WS** | **W** |  |
| ***Pseudomonas*** | 2756 | 1667 | 7381 | 25466 | 71 | 3582 | 564 | 750 | 11393 | 2442 | 8282 | 11788 | 4364 | 744 | 81250 |
| ***Bacillus*** | 769 | 106 | 363 | 8524 | 47 | 63 | 13429 | 365 | 149 | 45 | 247 | 1882 | 276 | 281 | 26546 |
| ***Clostridium*** | 1199 | 222 | 179 | 1194 | 2 | 30 | 3 | 59 | 20 | 1 | 21 | 737 | 2 | 3 | 3672 |
| ***Legionella*** | 18 | 101 | 7180 | 154 | 8 | 316 | 6 | 68 | 266 | 8 | 5720 | 56 | 118 | 3478 | 17497 |
| ***Acinetobacter*** | 448 | 10 | 157 | 45 | 49 | 18 | 56 | 9 | 165 | 37 | 92 | 1161 | 2 | 23 | 2272 |
| ***Aeromonas*** | 1 | 11 | 22 | 30 | 0 | 338 | 0 | 3 | 21 | 7 | 41 | 53 | 1 | 3 | 531 |
| ***Burkhoderia*** | 2 | 4 | 41 | 12 | 0 | 3 | 0 | 0 | 5 | 0 | 113 | 5 | 0 | 3 | 188 |
| ***Staphylococcus*** | 1 | 2 | 2 | 2 | 25 | 0 | 0 | 1 | 1 | 0 | 4 | 0 | 1 | 1 | 40 |
| ***Streptococcus*** | 1 | 2 | 2 | 2 | 25 | 0 | 0 | 1 | 1 | 0 | 4 | 0 | 1 | 1 | 40 |
| ***Corynebacterium*** | 35 | 11 | 14 | 10 | 0 | 7 | 1057 | 0 | 15 | 1 | 12 | 70 | 2 | 1 | 1235 |
| ***Coxiella*** | 0 | 0 | 0 | 0 | 0 | 0 | 4 | 0 | 6 | 0 | 0 | 0 | 0 | 0 | 10 |
| ***Escherichia*** | 5 | 10 | 88 | 968 | 1 | 11 | 2 | 20 | 35 | 1 | 82 | 12 | 47 | 5 | 1287 |
| ***Haemophilus*** | 0 | 0 | 0 | 0 | 11 | 0 | 0 | 0 | 2 | 0 | 0 | 0 | 0 | 0 | 13 |
| ***Moraxella*** | 0 | 0 | 3 | 0 | 1 | 0 | 0 | 0 | 2 | 0 | 0 | 0 | 0 | 6 | 12 |
| ***Erysipelothrix*** | 0 | 0 | 3 | 0 | 12 | 0 | 0 | 0 | 4 | 0 | 0 | 0 | 0 | 6 | 25 |
| **Total sequences** | **43036** | **75075** | **113743** | **155789** | **36465** | **104328** | **36900** | **22091** | **111458** | **26390** | **87537** | **66165** | **29132** | **25586** | **933695** |
| **TNSPP*** | **5235** | **2146** | **15435** | **36407** | **252** | **4368** | **15121** | **1276** | **12085** | **2542** | **14618** | **15764** | **4814** | **4555** | **134618** |
| **Percentage** | **12.2** | **2.9** | **13.6** | **8.7** | **0.7** | **4.2** | **41.0** | **5.8** | **10.8** | **9.6** | **16.7** | **23.8** | **16.5** | **17.8** | **12.2** |

*TNSPP = Total number of sequences belonging to potential pathogens
